# Assessing Bottlenose Dolphins’ (*Tursiops truncatus*) Health Status Through Functional Muscle Analysis, and Oxidative and Metabolic Stress Evaluation: A Preliminary Study

**DOI:** 10.3390/ani15091215

**Published:** 2025-04-25

**Authors:** Claudia Gatta, Eugenio Luigi Iorio, Carla Genovese, Barbara Biancani, Alessandro Mores, Daniele La Monaca, Chiara Caterino, Luigi Avallone, Guillermo J. Sanchez-Contreras, Immaculata De Vivo, Francesca Ciani, Simona Tafuri

**Affiliations:** 1 Department of Veterinary Medicine and Animal Production, University of Naples Federico II, Via F. Delpino 1, 80137 Naples, Italy; claudia.gatta@unina.it (C.G.); b.biancani@gmail.com (B.B.); chiara.caterino@unina.it (C.C.); avallone@unina.it (L.A.); stafuri@unina.it (S.T.); 2 Universidade Federal de Uberlândia, Faculdade de Medicina, Programa de Pós-graduação em Ciências da Súde, Rua Ceará - Umuarama, Uberlândia 38402-018, Minas Gerais, Brazil; eugenioluigi.iorio@gmail.com; 3 Zoomarine, Via dei Romagnoli, 00071 Torvaianica, Italy; cgenovese@zoomarine.it (C.G.); dlamonaca@zoomarine.it (D.L.M.); gsanchez@thedolphinco.com (G.J.S.-C.); 4 The Dolphin Company, Banco Chinchorro 87, Cancùn 77504, Quintana Roo, Mexico; 5 Department of Epidemiology, Harvard T. H. Chan School of Public Health, Boston, MA 02115, USA; nhidv@channing.harvard.edu

**Keywords:** *Tursiops truncatus*, oxidative stress, marine mammals, animal well-being, common bottlenose dolphin, tissue damage, metabolic stress

## Abstract

Oxidative stress (OS) occurs when there is an imbalance between the production of reactive oxygen species (ROS) and the body’s antioxidant defenses, causing damage to lipids, proteins, and DNA. In marine mammals, physiological adaptation to aquatic life conditions, such as prolonged and repeated dives resulting in cycles of hypoxia followed by reperfusion, is associated with increased production of ROS. This study examines the relationship between oxidative stress, muscular stress, and metabolic damage in the blood serum of eleven captive bottlenose dolphins (*Tursiops truncatus*), six males and five females. This relationship is investigated using oxidative stress markers (d-ROMs, OXY, and Oxidative Stress index, OSi) and biochemical parameter measurements, including glucose (GLU), aspartate aminotransferase (AST), creatine kinase (CK), and lactate dehydrogenase (LDH). Pearson’s sex correlation was performed, and males exhibited significantly higher pro-oxidant levels than females, suggesting a potential protective role of female hormones. Also, a positive correlation between pro-oxidants and antioxidants has been observed in relation to age, as older dolphins produced more ROS but also exhibited higher antioxidant capacity, likely to compensate for oxidative damage. Results show no significant correlation between biochemical parameters and oxidative stress markers. However, a moderately positive correlation between LDH and antioxidant (OXY) capacity was observed (r = 0.458), suggesting a possible association between tissue turnover and antioxidant defenses. The results indicate that the biochemical markers analyzed are not strong predictors of oxidative stress in bottlenose dolphins. However, the correlation between LDH and antioxidant capacity suggests that tissue turnover may affect antioxidant defenses. This is a preliminary study, and further research is needed to clarify these relationships in order to better understand physiological adaptations in dolphins and their implications for management, health, and welfare.

## Simple Summary

This study aims to assess the systemic pro-oxidant and antioxidant state of 11 common bottlenose dolphins (*Tursiops truncatus*) under human care, and to investigate the correlation between oxidative stress and biochemical parameters that indicate tissue damage or metabolic alterations. Blood aspartate aminotransferase, creatine kinase, lactate dehydrogenase, and glucose were evaluated as biochemical parameters. This study may elucidate the impact of oxidative stress on the metabolism and tissue function of the captive dolphin population. The management of marine mammals requires regular monitoring and blood assessment evaluation, in order to provide effective care and to promote their health and well-being.

## 1. Introduction

Oxidative stress (OS) is an imbalance between oxidizing and antioxidant molecules in favor of the oxidant, which is correlated with ageing and disease [1]. OS mechanisms are known as redox reactions [1]. Both oxidants and antioxidants are essential components in cellular processes, e.g., phagocytosis and maintaining the cell membrane, as well as physiological functions, such as immunological mechanisms and vascular function [2,3]. Oxidative damage, resulting from increased reactive oxygen species (ROS), is one of the main mechanisms by which the body can suffer both short- and long-term tissue damage. Excess ROS can cause inflammation, cellular apoptosis, and cell necrosis, contributing to the onset of chronic disease [4].

In marine mammals, such as non-captive populations of *Tursiops truncatus* (bottlenose dolphins), prolonged and repetitive dives cause cycles of hypoxia, followed by re-oxygenation upon surface ascent. This process favors the production of ROS, since the rapid increase in oxygen availability in the reperfusion phase can lead to the formation of free radicals [5,6]. During the dive, the animals experience bradycardia (reduction of heart rate) and peripheral vasoconstriction, which reduce oxygen consumption by less vital organs and preserve oxygen reserves in crucial organs like the heart and brain, as well as muscles with high levels of myoglobin [6,7,8,9]. Despite the potential threats of oxidative stress, cetaceans have developed complex antioxidant defense systems to minimize oxidative damage from apnea and deep diving [10].

Several biomarkers have been developed to assess the degree of OS and antioxidant capacity. Among these, the derivatives of reactive oxygen metabolites (d-ROMs) test is commonly used to measure levels of hydroperoxides, stable products resulting from the reaction of ROS with lipids and other biological molecules. The d-ROMs test provides a direct measure of an organism’s pro-oxidative state, reflecting the number of free radicals circulating [11,12,13], while the OXY-Adsorbent test evaluates the serum’s total antioxidant capacity by measuring its ability to neutralize a certain amount of oxidant. This test is essential to determine the effectiveness of the body’s antioxidant defenses and to understand how effectively the body can counteract the effects of ROS [12,13,14]. The Oxidative Stress index (OSi), which represents the ratio of ROS levels measured by the d-ROMs test and antioxidant capacity measured by the OXY-Adsorbent test, provides an overall measure of the body’s redox balance [15,16]. High OSi indicates an imbalance in favor of oxidants, suggesting increased oxidative stress and the risk of cellular damage.

The existing literature shows a correlation between OS and certain biochemical parameters, e.g., blood aspartate aminotransferase (AST), lactate dehydrogenase (LDH), and creatine kinase (CK), which are tissue damage indicators, and glucose (GLU) [15].

In marine mammals, the GLU fasting level is physiologically higher than in terrestrial mammals [17]. Medway and Geraci [18] suggest that this is a response to increased central nervous system glucose requirements during dives. Vallyathan et al. [19] hypothesize that higher glucose levels are needed to nourish the muscles during dives. *Tursiops truncatus* has postprandial hyperglycemia, which produces a prolonged glucose tolerance curve during 6–72 h of fasting [20]. This is comparable to type 2 diabetes in humans [20,21,22,23], but without the development of ketoacidosis, indicating unique physiological mechanisms of adaptation [22,24]. ROS can alter glucose regulation and compromise insulin sensitivity, contributing to insulin resistance and altering energy metabolism [25].

Aspartate Transferase (AST) is useful for assessing tissue activity and possible damage under OS conditions in both marine and terrestrial animals. ROS accumulation can compromise the integrity of liver and muscle cells, which causes the release of AST into the bloodstream [26]. AST is highly active in marine mammals, particularly in musculoskeletal and liver tissue [27], and, according to Ridgway [17], AST is comparatively higher in animals with higher metabolic rates.

LDH is an enzyme involved in anaerobic metabolism, and its release into the bloodstream is usually associated with tissue damage, especially in the muscles and liver [15,28]. OS can cause the release of LDH into the blood and act as an indicator of cell damage during hypoxia and reperfusion [29]. In cetaceans under human care, total serum LDH concentrations may be higher [27], and muscle hypoxia and perfusion phases during dives may stimulate an increase in ROS production, resulting in the release of LDH [30].

CK is a specific isoenzyme responsible for energy metabolism in muscles and is another important indicator of muscle damage. Its increase occurs during skeletal muscle injury, often associated with an intense activity, transportation, or stranding [27]. The intense muscle activity during swimming and diving, combined with the effect of ROS, can lead to a significant increase in CK, indicating muscle damage [31].

This preliminary study aims to evaluate biochemical parameters such as blood pro- and antioxidants, as well as blood levels of GLU, AST, LDH, and CK, that can help us to understand the state of animal health. We will not evaluate the effect of deep diving; rather, we will focus exclusively on oxidative stress among captive dolphins for management purposes. Such monitoring and blood testing are crucial for managing the dolphin population and implementing strategies to promote their health and well-being.

## 2. Materials and Methods

### 2.1. Animals and Sample Collection

For the present study, we used eleven clinically healthy dolphins (*Tursiops truncatus*), five females (F) and six males (M), aged between 5 and 42 years, housed in an Italian zoo (Zoomarine Italia, Torvaianica, Rome, Italy). The animals were considered healthy based on medical history and a regular physical examination carried out as part of the preventive medicine program. The dolphins were housed and treated in accordance with the Zoo Directive law (DL 73/2005), and blood samples were obtained according to D.M. 469/2001, which establishes the management objectives and requirements for keeping the species *Tursiops truncatus* under human care. All dolphins kept in the facility have been trained to participate voluntarily in veterinary and breeding procedures in order to ensure regular diagnostic analysis. All animals voluntarily presented their tail flukes for blood collection [32]. This study aimed to minimize any factors that could have affected the reliability of the values obtained and reported. The environmental conditions of the aquatic farm, where the dolphins are kept in captivity, are carefully controlled and consistent for all animals, as are their diet and programmed training activity. Therefore, these factors did not impact the reliability of the data. Upon clinical examination, the dolphins presented no clinically relevant symptoms, signs of inflammation, or health abnormalities, and exhibited a mesomorphic body condition score. The complete blood count (CBC) values and clinical biochemistry markers were within normal ranges. Animals with altered health conditions were excluded from the study. All animals were fasted at the time of blood collection, in order to avoid any inconsistency that might affect the results. Blood samples were collected in single tubes with separator gel, centrifuged at 3000× *g* for 10 min to obtain blood serum, and then stored at −20° until analysis (within one month of collection).

### 2.2. Biochemical Analyses

Blood serum biochemical analyses of 11 bottlenose dolphins were conducted using an automatic clinical chemistry analyzer validated for marine species, the Clinical Chemistry Automatic PKL PPC 125 (POKLERITALIA 125, Salerno, Italy). The following parameters were measured: Glucose (GLU) measured in mg/dL, to assess blood sugar levels; aspartate aminotransferase (AST) measured in U/L, to evaluate liver and muscle function; creatine kinase (CK) measured in U/L, to assess muscle damage; and lactate dehydrogenase (LDH) measured in U/L, to detect tissue damage.

### 2.3. Measurement of Oxidative Status

The pro-oxidative status was determined by the d-ROMs test, which determines the level of OS by measuring the amount of organic hydroperoxide that oxidizes N,N-diethyl-p-phenylenediamine, resulting in a pink-colored derivative photometrically quantified at 505 nm [12,13,33]. According to Lambert–Beer’s law, the intensity of the developed color is directly proportional to the concentration of ROMs and is expressed as Carratelli Units (1 CARR U = 0.08 mg hydrogen peroxide/dL).

The antioxidant barrier was evaluated using the OXY-Adsorbent test, which measures the ability of samples to counteract the oxidation induced by a solution of hypochlorous acid (HClO). Unreactive HClO radicals react with the chromogen solution of N,N-diethyl-p-phenylenediamine, producing a colored complex measured at 505 nm. The results were expressed as μmol HClO/L [12,13,33]. All OS kits were purchased from Diacron International, Grosseto, Italy.

The ratio between the values of d-ROMs and OXY (× 100) (OSi) is an arbitrary value, used as an index of plasma redox status. High values indicate a higher concentration of oxidized molecules than non-enzymatic antioxidants [16].

### 2.4. Statistical Analysis

We used Pearson correlation analysis to evaluate the relationship between the sex and age of animals and the oxidative stress markers. Pearson correlation coefficients were calculated to assess the relationships between the biochemical parameters (GLU, AST, CK, LDH) and the levels of oxidative stress markers. Correlations were interpreted in terms of the strength and direction of the relationship between the parameters.

A multiple linear regression analysis was conducted to assess the combined influence of biochemical parameters (GLU, AST, CK, LDH) on oxidative stress markers. The dependent variable for each regression was one of the OS measures, while the independent variables were the four biochemical parameters.

The statistical analyses were performed using R software (version 4.0.3). Results were considered statistically significant at *p* < 0.05.

## 3. Results

The results of the oxidative status marker measurement in the eleven dolphins examined are shown in Table 1.

### 3.1. Correlations Between Sex and Oxidative Stress Markers

Table 2 reports the correlations between the sex of the animals and oxidative stress markers.

The results indicate a moderate positive correlation between sex and d-ROMs values (r = 0.459) and OSi (r = 0.390). The correlation between sex and OXY values was very weak (r = 0.122).

### 3.2. Correlation Between Age and Oxidative Stress Markers

The analysis of correlations between age and oxidative stress markers is shown in Table 3. The results show a moderate positive correlation between age and OXY levels (r = 0.468) and a weaker correlation between age and d-ROMs levels (r = 0.328). The correlation between age and OSi was negligible (r = 0.148).

### 3.3. Correlations Between Biochemical Parameters and Oxidative Stress Markers

The values for GLU, AST, CK, and LDH reported in Table 4 were within the normal range [27].

Correlation analysis did not reveal strong relationships between the biochemical parameters (GLU, AST, CK, LDH) and the OS markers (d-ROMs, OXY, OSi) (Table 5).

The pro-oxidants did not show a strong correlation with any of the biochemical parameters. The most relevant result was a negative, but not significant, correlation with CK (r = −0.390), indicating a potential link between lower muscle enzyme levels and reduced OS.

A moderately positive correlation was observed between LDH and OXY (r = 0.458), suggesting a potential relationship between tissue turnover, as indicated by LDH levels, and the body’s total antioxidant capacity.

OSi showed no significant correlations with any of the biochemical parameters. The correlations for OSI with GLU (r = 0.175) and CK (r = −0.248) were weak.

### 3.4. Multiple Linear Regression Analysis

Multiple linear regression analysis was performed to examine the combined effects of biochemical parameters (GLU, AST, CK, LDH) on d-ROMs, OXY, and OSi (Table 6).

The results show no significant differences between biochemical parameters and d-ROMs values, although CK showed a negative trend (*p* = 0.228), indicating a potential link between muscle enzyme levels and OS.

The regression model for OXY showed that LDH was close to significance (*p* = 0.058), suggesting a possible association between tissue turnover and antioxidant capacity.

Finally, the regression model for OSi showed no significant difference among the biochemical parameters.

## 4. Discussion

Some parameters related to oxidative stress and clinical biochemistry were measured in blood serum samples of *Tursiops truncatus* (bottlenose dolphins) living under human care at Zoomarine in Rome. The values were analyzed to assess the health and well-being of the study animals.

Sex analysis showed a moderately positive correlation for d-ROMs and OSi levels in males, suggesting that males may have a higher pro-oxidative state than females. Males may have a higher basal metabolism than females, associated with increased production of ROS during metabolic processes [34].

In addition, the competitive and territorial behavior of males may require higher energy consumption, contributing to a higher oxidative load [35]. Studies on other marine species, such as pinnipeds in the wild, have shown that males exposed to social stress or competition exhibited a significant increase in ROS levels [35], a phenomenon that may be similar in dolphins. Sex hormones can also affect the redox state. Testosterone, which is predominant in males, has been associated with increased ROS production and reduced antioxidant activity [36]. In contrast, estrogen has antioxidant properties that can protect females from the negative effects of OS [37]. In addition, studies on terrestrial animals have shown that males tend to show a higher susceptibility to OS during periods of increased reproductive activity, a phenomenon which may also be relevant in marine mammals [38]. A weak correlation was observed between sex and OXY levels (r = 0.122), which may indicate that intrinsic antioxidant defenses do not vary significantly between the sexes. In females, estrogen offers significant protection due to its antioxidant properties, directly neutralizing free radicals and limiting oxidative damage [37]. This could play a very important role during the pregnancy and lactation phases, when females must maintain a stable physiological balance to preserve energy resources needed by their offspring. Studies on wild elephant seals have shown that males are subject to greater OS during periods of reproductive competition, because they have a higher energy expenditure and frequent conflicts for social competition [39]. Similarly, during the breeding season, male birds also show higher testosterone production and higher levels of ROS than females [40].

The observation of a moderate positive correlation between age and OXY levels suggests that older animals have developed a physiological adaptation to enhance their antioxidant defenses and maintain a stable redox balance, as hypothesized by Giorgi et al. [41] in terrestrial mammals. These findings contrast with the traditional idea of a progressive decline in antioxidants with advancing age and raise questions for further study. Long-lived marine mammals can develop physiological strategies to counteract the cumulative effects of OS. This phenomenon has also been observed in wild cetaceans, with older individuals showing higher levels of antioxidant enzyme activity [42]. The antioxidant activity of long-lived land animals, such as elephants and turtles, has been shown to increase with age, which is similar to the adaptations observed in marine mammals in our study [43]. There was a moderate correlation between age and the extent of the antioxidant barrier in the dolphins we studied, though the small sample size may account for this result.

Although d-ROMs levels show a weak positive correlation with age, the increase in oxidative load is compensated by an increase in antioxidant capacity. This balance is confirmed by the negligible correlation between age and OSi, which suggests effective physiological compensation. Similar results have been observed in marine organisms under environmental stress conditions, such as temperature changes or hypoxia [44]. The analysis of the biochemical values GLU, AST, CK, and LDH performed in the present study yielded results within normal limits for bottlenose dolphins [27]. Our study found no significant correlation between the main biochemical markers (GLU, AST, CK, and LDH) and the OS markers (d-ROMs, OXY, OSi). The absence of significant correlations between biochemical parameters and OS markers suggests that, in the captive dolphins of our study, OS levels are not strongly influenced by metabolic and tissue damage parameters. Wild marine mammals have an extraordinary ability to adapt to extreme environmental conditions, such as long dives. During dives, transient hypoxia followed by surface tissue reperfusion increases ROS production [45,46,47,48]. Also, dolphins have developed powerful antioxidant defense systems to counteract the excessive production of ROS. Enzymes such as SOD, catalases, and glutathione peroxidases play a crucial role in this defense [35,49]. These defenses may be effective in preventing biochemical markers of tissue damage. In addition, during dives, dolphins reduce oxygen consumption through physiological mechanisms such as bradycardia and peripheral vasoconstriction, which limit the supply of blood and oxygen to less vital tissues [7]. This phenomenon, combined with the presence of high levels of myoglobin in muscles, helps to reduce ROS damage during dives. This could explain the weak correlation between OS markers and tissue damage in marine mammals and other animals [8,9,45]. Previous studies have shown that the gastrointestinal tract of bottlenose dolphins plays a crucial role in regulating metabolism and maintaining homeostasis, with leptin-like peptides such as orexin and nesfatin-1 likely influencing glucose metabolism [50,51,52] and thus potentially intervening in the OS balance. Nesfatin-1 has been linked to the regulation of insulin sensitivity and glucose metabolism, playing a potential role in stabilizing blood sugar levels [21,52], and potentially affecting the oxidative balance in dolphins. This suggests that the glucose metabolism in dolphins may also be regulated by gastrointestinal endocrine factors, which influence systemic oxidative and energy balance, as observed in other mammals. The biochemical parameters measured in this study (GLU, AST, CK, LDH) could be sufficiently sensitive or specific indicators of ROS damage in dolphin populations under human care. Multiple regression analysis confirmed the absence of significant relationships between biochemical parameters and OS indicators. However, LDH showed a trend towards significance with OXY levels (*p* = 0.058), suggesting a possible link between tissue turnover and antioxidant capacity.

LDH is an enzyme involved in anaerobic metabolism, and its presence in the blood is often associated with tissue damage, especially in muscles and the liver [15]. We can therefore assume that increased LDH levels may reflect an increase in cell turnover or tissue repair in response to oxidative damage [29].

CK, an indicator of muscle damage, showed a negative trend with d-ROMs and OXY, although this was not significant. This may indicate that muscle damage (measured by CK) is not strongly associated with the production of ROS in dolphins, or that antioxidant defense mechanisms are sufficient to prevent an increase in OS in response to muscle damage [6,8,9].

The reported data, while intriguing, are applicable to captive dolphins only, as there are understandable limitations that do not allow for broader application to wild dolphins. This study should also be considered preliminary due to the limited number of animals examined, which reflects the difficulty in obtaining samples from this species, given the small number of dolphins under human care. Despite these limits, we are confident that the knowledge gained could be useful in more precisely defining the ranges of measured oxidative stress markers. Furthermore, it provides insights into the redox status of the animals and could potentially allow for targeted interventions to restore the balance between pro- and antioxidants. Additionally, it would be beneficial to offer potential directions for further research, particularly regarding non-invasive methods to assess oxidative stress in both captive and wild dolphins. The ability of dolphins to manage OS through antioxidant defense systems may not be sufficient under increased stress conditions, such as pollution, environmental changes, or intense physical activity. Monitoring OS levels and biochemical biomarkers could be a valuable tool to assess the general health status of individuals and optimize species management and conservation strategies. In particular, in the fields of veterinary care and dolphin welfare under human care, these tests could be integrated into routine protocols to identify oxidative imbalances early. Thus, it would be possible to make changes to the environment and physical activity, as well as add antioxidant foods to the diet.

## 5. Conclusions

The use of specific biomarkers for OS, measured with the d-ROMs test and the OXY-Adsorbent test, allows us to assess the redox state of individuals and to intervene early in case of oxidative imbalance. These tools can be particularly useful for detecting the first signs of OS in captive populations or degraded natural conditions. Although captive bottlenose dolphins show a remarkable ability to manage OS through antioxidant defense systems, such mechanisms may not be sufficient in high-stress conditions, such as pollution, environmental changes, or intense physical activity. Regular monitoring of OS levels and biochemical biomarkers could be a useful tool to assess the general state of health of individuals. Further investigation with a larger sample size of dolphins, both in the wild and in captivity, would be valuable for adapting conservation programs for this species and minimizing the risks associated with oxidative stress, especially under increased environmental stress. Furthermore, such research could support efforts to prevent health deterioration and improve the well-being of bottlenose dolphins.

## Figures and Tables

**Table 1:** Oxidative stress markers in 11 dolphins at different ages and sexes.

Animals	Age	Sex	d-ROMs (U. Carr)	OXY (μmol HClO/L)	OSi (d-ROMs/OXY)
**1**	42	M	80	1332.5	6
**2**	32	M	75	1259.5	5.9
**3**	5	F	81	1251.0	6.5
**4**	19	F	61	1264.6	4.8
**5**	9	M	82	1145.4	7.1
**6**	26	M	91	1255.6	7.2
**7**	25	M	78	1280.2	6.1
**8**	19	F	84	1268.4	6.6
**9**	12	F	67	1268.2	5.3
**10**	7	F	58	1238.5	4.7
**11**	14	M	69	1348.6	5.1

**Table 2:** Pearson’s correlation coefficients between sex and oxidative stress markers.

Variable	Sex Pearson’s Coefficient (r)
**d-ROMs** (U.Carr)	0.459
**OXY** (µmol/L)	0.122
**OSi** (d-ROMs/OXY)	0.390

**Table 3:** Pearson’s correlation coefficients between age and oxidative stress markers.

Variable	Age Pearson’s Coefficient (r)
**d-ROMs** (U.Carr)	0.328
**OXY** (µmol/L)	0.468
**OSi** (d-ROMs/OXY)	0.148

**Table 4:** Biochemical parameters analyzed in dolphins.

Animals	GLU (mg/dL)	AST (U/L)	LDH (U/L)	CK (U/L)
**1**	135.9	354.4	798	99
**2**	91.3	174.5	479	122
**3**	114.4	154.1	2395	58
**4**	111.5	163.4	319	104
**5**	124.3	240.1	160	151
**6**	106.1	218.1	479	86
**7**	142.4	221.4	798	140
**8**	108.9	157.6	319	31
**9**	111.9	370.8	160	68
**10**	107.8	153.6	479	188
**11**	108.6	199.6	319	80

**Table 5:** Correlation analyses between biochemical parameters and oxidative stress markers.

Biochemical Parameter	Correlation with d-ROMs (r)	Correlation with OXY (r)	Correlation with OSi (r)
**GLU**	0.195	0.075	0.175
**AST**	0.081	0.193	0.030
**CK**	−0.390	−0.394	−0.248
**LDH**	0.143	0.458	−0.012

**Table 6:** Multiple regression between biochemical parameters and oxidative stress markers.

Biochemical Parameter	Coefficient (d-ROMs)	*p*-Value (d-ROMs)	Coefficient (OXY)	*p*-Value (OXY)	Coefficient (OSi)	*p*-Value (OSi)
**GLU**	0.1608	0.632	−0.9205	0.484	0.0190	0.546
**AST**	−0.0127	0.820	0.1009	0.645	−0.0015	0.779
**CK**	−0.1136	0.228	−0.6523	0.094	−0.0058	0.491
**LDH**	0.0094	0.646	0.1754	0.058	−0.0002	0.908

## Data Availability

All the data are available on request to the first author.
